# Interventional radiology training: where will technology take us?

**DOI:** 10.1259/bjro.20190002

**Published:** 2019-08-16

**Authors:** Meghavi Mashar, Andrew Nanapragasam, Philip Haslam

**Affiliations:** 1 Oxford University Hospitals NHS Foundation Trust, Oxford, United Kingdom; 2 King’s College Hospital, London, United Kingdom; 3 The Newcastle Hospitals NHS Foundation Trust, Newcastle-upon-Tyne, United Kingdom

## Abstract

Interventional radiology is a relatively young specialty, and it is undergoing a period of considerable growth. The benefits of a minimally invasive approach are clear, with smaller incisions, less pain, and faster recovery times being the principal benefits compared to surgical alternatives.

Trainees need to acquire the technical skills and the clinical acumen to accurately deliver targeted treatment and safely follow up patients after the procedure. The need to maintain an efficient interventional radiology service whilst also giving sufficient time for trainee education is a challenge. In order to compensate for this, novel technologies like virtual reality (VR), augmented reality (AR), cadaveric simulation, and three-dimensional (3D) printing have been postulated as a means of supplementing training.

In this article, we outline the main features of these innovative strategies and discuss the evidence base behind them. Benefits of these techniques beyond pure clinical training include the standardization of educational cases, access to training at any time, and less risk to patients. The main disadvantage is the large financial outlay required. Therefore, before widespread uptake can be recommended, further research is needed to confirm the educational benefit of these novel techniques, both in and of themselves and in comparison to existing clinical-based education.

Interventional radiology is a young speciality, with relatively few consultant practitioners providing a service that is increasingly in demand. The paucity of consultants makes training a new generation of interventional radiologists a logistical challenge. Becoming a competent interventional radiologist requires knowledge in basic clinical sciences such as anatomy and pathology, as well as procedural and clinical skills as outlined by professional bodies, such as the Royal College of Radiologists and the Cardiovascular and Interventional Radiological Society of Europe. The acquisition of these skills has typically relied upon an apprenticeship model, with trainees learning from supervised practice on real clinical cases. Whilst this method has been the long-established standard, it is not beyond critique.

The foremost concern with real world apprenticeship training is the risk to patients in having trainees learn their craft in the clinical setting. There is also the issue of the non-uniformity of case mix exposure amongst trainees learning on the job, leading to a potentially marked difference in competence and confidence. In addition, the time constraints imposed by turnaround targets may compromise education as sufficient time cannot always be afforded to teaching juniors.^1^

Innovative technologies have great potential in healthcare and have already taken root in training. For example, electronic log books, electronic portfolios and mobile learning applications are already an essential part of training. Moreover, traditional methods for acquisition of knowledge are being substituted by e-learning modules such as the Radiology-Integrated Training Initiative, which also provides a means of tracking trainee progress. With regards to technical competence, simulation has increasingly been incorporated into surgical curricula as a non-invasive, standardized, and time-efficient means of trainee education. Although initially devised for surgical education, the principle of simulation training can be applied to interventional radiology.

When compared to surgical operations, interventional image-guided procedures are less invasive, have a shorter recovery time, and a lower cost. For these reasons, interventional radiology is a specialty that is rapidly growing. One might expect the increased volume to correspond to more training opportunities. However, advances in diagnostic imaging techniques have meant that “easier” interventional techniques, like diagnostic angiography, are being supplanted by MRI and CT imaging, with interventional radiology focussing on the more technically advanced procedures. The reduction in the availability of these easier cases, means trainees miss out on an important learning opportunity.

Simulation methods available to interventional radiologists range from plastic models to virtual environments to cadaveric simulation. This review aims to capture the recent advances in training and their possible uses and challenges for interventional radiology training.

## Cadaveric simulation training

Cadaveric simulation has been used extensively in multiple surgical specialities. Advantages of cadaveric simulation include realistic anatomical portrayal, capturing anatomical variants and pathology. However, cadaveric models have been criticised as post-mortem tissue is not a completely accurate facsimile for live tissue. In an effort to combat this, soft-fix cadavers (embalmed by the Thiel method) have gained popularity for their better textural accuracy.^2^ Despite these improvements, live tissue remains discernibly different in its texture and response characteristics. However, while live animal models may provide realistic tissue, they do not replicate human anatomy. Furthermore, special ethical permissions are necessitated prior to the use of anesthetized animal models.^3^ For these reasons, the human cadaveric model is generally preferred in the existing surgical literature.

Certain centres are beginning to utilize cadavers for interventional simulation purposes. In the USA, Meek et al trialled lightly embalmed cadavers to practice ultrasound-guided vascular line placement.^4^ A further workshop was held on ultrasound-guided liver and kidney biopsies. Subjects reported high levels of satisfaction and an increase in self-reported confidence following the training exercise.

McLeod et al^5^ recently devised a method to make a functional cadaver using extracorporeal aortic perfusion to provide pulsatile antegrade flow. While it is not possible to quite replicate the high pressures in real human anatomy (due to the lack of vessel muscle tone), this model still provides a physiologically robust simulation for endovascular intervention. In an effort to further improve the realism of simulation training, the creators behind the *SimLife* model successfully managed to add respiratory and hemodynamic control to a cadaver.

The use of cadaveric simulation training is a realistic means for training interventional radiologists. Despite the benefits, the financial outlay required for the purchase and maintenance of simulation equipment does limit its large-scale implementation. In addition, prospective centres have to establish rigorous ethical standards to ensure the cadavers are treated appropriately.

## Virtual Reality & Augmented Reality

VR learning environments allow a trainee to interact with objects in an entirely computer-generated 3D space. Whereas AR incorporates virtual components into a real environment.

VR has been demonstrated to be a useful resource for the learning of anatomy. At the University of Manchester, students using an open source software, *Blender*, were found to have a comparable anatomy knowledge to those taught by traditional methods of cadaveric dissection and textbooks.^6^ Moreover, another study indicated medical students had greater answering speeds in anatomy assessments, greater rates of satisfaction with the learning methods, and a greater enthusiasm for their future careers when compared to conventional methods.^7^ Lastly new 3D VR headsets such as the *Microsoft Hololens* show potential for real-time pathology*–*radiology correlation and evaluation of gross pathology.^8^

Interventional radiologists are increasingly taking ownership of the patient’s post-procedural care as evidenced by the creation of IR ward rounds and clinics. Virtual ward rounds and virtual patient encounters may show promise as a tool for training learners. Storck et al^9^ have created a web-based software where users can examine virtual patients, make diagnoses and order investigations. This may enable the development of medical decision-making skills in both clinics and post-operative settings. Additionally, virtual ward rounds may become a routine part of NHS service for post-operative patient follow up. A recent systematic review^10^ has demonstrated that patients with heart failure, have reduced rates of re-admission and mortality with post-discharge ward rounds than with standard community-based care. Hence not only may virtual clinical encounters prove educationally beneficial, but they may also be more representative of the future health service.

Magee et al developed an AR simulator as early as 2007 for ultrasound-guided intervention with a mannequin, a mock ultrasound probe, and a needle incorporated into a virtual ultrasound environment.^11^ Validation was undertaken by 60 radiologists who felt the simulator did accurately replicate the technique but the haptic feedback was limited, as this was not incorporated into the simulator.

Coles et al^12^ developed *PalpSim*, an augmented reality simulator to teach arterial puncture. The trainee can view a computer-generated patient and needle, which they can interact with using their own hands, and receive haptic feedback. This can overcome the limitations of material degradation seen with the repeated use of a physical mannequin. In addition, the computer generated model can be modified to simulate different structures. During validation of this model, practitioners reported that picking up and inserting the needle felt realistic, however, the feeling of free space was thought to be distorted. The authors propose adjustments to hardware, which they believe would rectify this. Further realism can be added with VR generated respiratory motion, as shown in the Johnson et al study, where respiratory movement was incorporated into a virtual liver biopsy environment.^13^

Simulators can also be used for crisis preparation for all members of a team, allowing training and assessment of both practical and non-practical skills. One group developed a ruptured abdominal aortic aneurysm and endovascular repair simulation using two VR simulators in an angiosuite.^14^ Performance was assessed by recording the time to each step, with experienced clinicians performing better than more junior trainees.

The work described above showcases the breadth of opportunity and benefits of VR and AR in interventional radiology training. However, these modalities are yet to find a definitive place in most training schemes. One of the major challenges for designers of virtual environments, is the need to create accurate haptic feedback and believable virtual environments. Over time, we have seen progressive technological refinements that have increased the realism of the simulation, and presumably the learner’s experience. However, one review demonstrated no significant gains in the difference between high fidelity and low-fidelity simulators for a particular simulation.^15^ The authors postulated that this unexpected finding may be because novices could not effectively understand the additional cognitive load provided by a high-fidelity simulator. Clearly, there is a balance to be struck between realism and information overload. Exactly where this line lies is hard to define. The authors of the study suggest that a model of progressive fidelity may be advantageous, where trainees can use more complex simulators as they progress through training.

## 3d printing

3D models of CT or MRI are another medical innovation with potential for education and enhanced operative practice.^16^ Mafeld et al created a 3D model of the aorta to simulate endovascular tasks.^17^ The simulator was evaluated by 96 candidates who reported a largely favourable experience. In another study, trainees were randomised into two groups, with both groups given a CT angiogram of an abdominal aortic aneurysm. One of the groups also received a 3D model generated from the CT imaging. The group with a model scored higher on questions centred around pre-operative planning, indicating a role for 3D models in improving visuospatial skills for novice interventionalists.^18^ Compared to the other techniques, 3D printing is relatively inexpensive and requires less technological maintenance.

## Conclusions

Cadaveric simulation, virtual/augmented reality environments, and 3D printing in interventional radiology have demonstrated promise as a means of medical education. Despite the potential for benefit, their use is rare in UK radiology training schemes. This may be because of concerns around the transferability of simulation acquired skills into real world clinical competence. Designing studies to prove that use of novel simulation techniques translates into actual benefits will be challenging. However, this work is essential to convince educationalists of the benefits of simulation training. In addition, the evidence base will have to be compelling given the significant financial outlay required for creation and continued running of these simulation suites.
